# De-escalation of the Agitated Pediatric Patient: A Standardized Patient Case for Pediatric Residents

**DOI:** 10.15766/mep_2374-8265.11388

**Published:** 2024-03-08

**Authors:** Adam Kronish, Daniel Alanko, Victoria R. Quinn, Charles Wulff, Elizabeth Stone, Robyn Wing

**Affiliations:** 1 Second-Year Fellow, Division of Adolescent Medicine, Children's Hospital of Philadelphia; 2 Second-Year Fellow, Department of Pediatric Emergency Medicine, Children's Hospital of New Jersey at Newark Beth Israel Medical Center; 3 First-Year Fellow, Department of Pediatric Emergency Medicine, Hasbro Children's Hospital; 4 Attending Psychiatrist, Department of Psychiatry and Behavioral Sciences, Boston Children's Hospital and Harvard Medical School; 5 Primary Care Pediatrician, Independent Practice; 6 Associate Professor, Division of Pediatric Emergency Medicine, Departments of Emergency Medicine and Pediatrics, Warren Alpert Medical School of Brown University and Rhode Island Hospital/Hasbro Children's Hospital; Director of Pediatric Simulation, Lifespan Medical Simulation Center

**Keywords:** Agitation, Child and Adolescent Psychiatry, Pediatric Mental Health, Pediatrics, Psychiatry, Simulation

## Abstract

**Introduction:**

Over the past 5 years, pediatric mental health emergencies requiring emergency safety evaluations and inpatient boarding of pediatric patients requiring psychiatric admission have increased. Pediatric trainees must learn to effectively and safely de-escalate a patient with agitated or aggressive behavior, as mental health patients take up a larger proportion of their patient population. This standardized patient case addresses gaps in knowledge and skills to ameliorate the care of children and adolescents with behavioral crises in the hospital.

**Methods:**

Resident learners were presented with a teenage patient admitted to the hospital and awaiting inpatient psychiatric placement for suicidal ideation who became acutely agitated with aggressive behaviors. Learners were expected to attempt to verbally de-escalate the patient and select an appropriate pharmacologic agent for decreasing agitation in the patient. A standardized debrief was conducted with the assistance of child and adolescent mental health experts.

**Results:**

Twenty-two learners participated in this activity. Residents' confidence in their management skills of the acutely agitated pediatric patient significantly increased after completion of the activity. Seventy-three percent of learners felt confident or very confident in their de-escalation skills at the end of the case, and 86% agreed that the case improved their confidence in managing acute agitation scenarios on the inpatient wards.

**Discussion:**

This case led to overall increased self-efficacy in caring for the acutely agitated pediatric patient. Future iterations may include multidisciplinary learners of various skill levels and evaluating changes in patient-centered outcomes, such as restraint use, after implementation of the case.

## Educational Objectives

By the end of this activity, learners will show increased confidence in being able to:

1. Assess situational safety for a patient, staff, or property.
2. Manage an acutely dysregulated patient independently.
3. Identify when a pharmacologic option is indicated for patients with acute anxiety, agitation, or aggression.
4. Select an initial medication for a child in acute psychiatric decompensation based on the individual needs and history of the specific patient.

## Introduction

In October 2021, the American Academy of Pediatrics, the American Academy of Child and Adolescent Psychiatry, and the Children's Hospital Association released a joint statement declaring a national emergency in pediatric mental health.^1^ As hospitalizations for suicidal ideation and other serious mental health issues have been increasing for well over a decade,^2^ patients commonly require admission to the general pediatric ward to board while awaiting placement to an inpatient psychiatric bed. A 2020 review found that as many as 58% of psychiatric patients experience boarding, with 26%–49% boarding in inpatient units.^3^ Patients board for an average of 48–72 hours before placement, and these stays continue to grow even longer.^4^

Boarding produces challenges for both patients and the providers caring for them, who are often not trained in psychiatric care. For example, children admitted as psychiatric boarders are less likely to receive counseling and psychiatric medications compared to patients admitted directly to psychiatric units.^5^ Furthermore, several studies have identified a reverse triage system where patients with higher psychiatric severity are more likely to board inpatient.^6^ This creates an environment where escalations due to dysregulated behavior occur more frequently,^7^ so providers should be trained on how to manage these situations to ensure the safety of patients and hospital staff.

At our institution, pediatric residents on hospitalist teams are the first-line providers responsible for these patients' care. The above challenges coupled with long-standing deficiencies in resident education regarding mental health issues have created obstacles to quality psychiatric patient care.^8^ Only a minority of pediatric trainees report high levels of perceived competence in the assessment and treatment of mental health conditions.^9^ Training in the stabilization of severe psychiatric illness is an important skill for future pediatricians caring for patients with complex mental health needs.

A mental health curriculum for pediatric trainees was recently published in *MedEdPORTAL,* but it largely focuses on two educational tools: interactive lecture-style conferences and case-based sessions.^10^ Simulation is a validated tool that could augment such a curriculum to teach learners effectively how to manage acute psychiatric patients in a safe environment.^11,12^ While simulation has been described as a vehicle for teaching pediatric residents about mental health issues,^13^ its utility in managing acutely aggressive pediatric patients has not. Some work has been done with simulated behavioral de-escalation in the emergency department, but to our knowledge, there is none directed at pediatric providers.^14^ Here, we present a case of acute psychiatric illness in an admitted pediatric patient, with pediatric trainees as the target learners. We chose a standardized patient (SP) case as the teaching method to immerse the learner and evoke authentic emotional and physical reactions to this difficult scenario. Learners were encouraged to develop strategies for managing a verbally aggressive patient. Afterwards, an expert in pediatric mental health joined for a debrief. Additionally, this case provided an opportunity for residents to discuss the indications and efficacy of other de-escalation tools, including physical restraints, psychoactive medications, and the consultation of psychiatric providers. This was primarily a low-stakes formative teaching activity for pediatric residents integrated into a nongraded longitudinal didactics curriculum.

## Methods

### Development

Pediatric senior residents, a pediatric emergency medicine (PEM) physician, and a child and adolescent psychiatrist developed this adaptable SP case as part of an inpatient-based pediatric simulation curriculum for residents rotating on the pediatric medical wards. Prerequisite knowledge included knowledge of psychotropic medications, such as antihistamines, benzodiazepines, and antipsychotics, though dosing knowledge was not required. The American Academy of Pediatrics guidelines by Chun and colleagues and other resources outlined specific verbal de-escalation techniques and provided tables of medications used in agitation for those who wanting to supplement or precede this learning activity.^15,16^ Five appendices were created for this case's implementation: a facilitator guide (Appendix A), a debriefing guide (Appendix B), a learner survey (Appendix C), a critical action checklist (Appendix D), and an SP guide (Appendix E).

Please note that the word *simulation* is used throughout this report and in the appendices, survey items, and other aspects of this resource to refer to the SP case because the educational activity was a part of an active-learning curriculum containing the case as well as multiple simulation cases. The original surveys were written to be standardized across the curriculum. We recognize that *SP activity/case* would be more accurate to describe this educational activity.

### Equipment/Environment

The case's setting at our institution was half of a multipurpose room set up to simulate a patient room with a patient bed (or table with sheets and pillows to appear as a bed). The scenario could be run in a dedicated simulation center or an empty patient room, although possible limitations of using an empty patient room on the clinical floors are discussed below. Vital signs were available if requested although they were not necessary to the case. No preparatory materials were provided to learners prior to the activity.

### Personnel

Personnel included a senior resident or faculty as a facilitator; SPs and/or embedded participants to play the patient, a nurse, and the patient's parent; and a child/adolescent psychiatry specialist as a content expert for debriefing. If available, an SP with training according to standards should play the role of the patient.^17^ We recruited our SP, who had prior experience with behavioral health simulations, from our simulation center. The SP was given the case script prior to the session and had practiced the role with facilitators prior to participant arrival. If limited SPs are available, the role of the patient's parent can be omitted. Between three to six learners could participate in the case at once.

### Implementation

The case was planned during scheduled didactic time for residents working on the pediatric wards. Each educational session lasted 30 minutes, with 10–15 minutes for the simulated case and the remaining 15–20 minutes for debriefing. Each session included three to six learners, with two to three actively participating (at least one volunteer was patient facing, while another communicated with other members of the team for resource and medication management) and the remainder observing the case and then actively participating in the debrief. Facilitators used the critical action checklist (Appendix D) to quantify their observations of learners' actions for use in the debriefing. All participants and observers were in the debrief.

The facilitator guide (Appendix A) detailed the case fully. After a 2-minute prebrief to establish a safe learning environment and review confidentiality within the session, learners decided who would actively participate in the case and who would observe. Next, a facilitator provided context, informing learners that they had been called for a Code Grey (acute safety concern/behavioral code) on a patient under their care as the overnight residents. The residents entered the room to see the patient pacing and visibly agitated. Through effective verbal de-escalation and division of tasks, the learners de-escalated the patient and selected a medication for agitation to be offered to the patient. History from either the patient's parent or the bedside nurse revealed that the patient had a history of a paradoxical reaction to diphenhydramine. Therefore, learners needed to choose another pharmacologic agent for agitation. The case ended when the patient accepted an oral medication voluntarily or the learners attempted to administer an intramuscular medication.

### Debriefing

Immediately following the activity, the group facilitators and content experts led a debrief, as outlined in Appendix B, using a modified PEARLS format.^18^ The content experts included a child and adolescent psychiatry attending, pediatric psychiatry residents, and a pediatric psychologist. The debriefing guide also featured an abbreviated debriefing checklist, which was provided to facilitators to ensure major opportunities for learning and reflection, including verbal de-escalation strategies, medication selection, affective reactions to the case, and growth opportunities, would be discussed. At the end of the debrief, time was allotted for any further questions or reactions of learners. Finally, each learner was asked to highlight at least one individual take-home point that they had learned from their participation.

### Assessment

The session concluded by inviting participants to complete an anonymous online survey (Appendix C) regarding their perceptions of the case and its educational effectiveness. The survey was developed by the authors, using 5-point Likert-scale questions regarding participant comfort with achieving the learning objectives of the case. The single survey was administered after completion of the debrief and consisted of retrospective questions regarding confidence prior to completing the case (pretest item 4 of Appendix C) and questions inquiring about confidence around the content of the case after completion of the activity (posttest item 13 of Appendix C).

Program evaluation questions were embedded within the survey, including items 5–9 and the open-ended questions. Inference was based on the *t* distribution, estimated using generalized linear mixed-effects modeling to account for the correlation between pretest (item 4) and posttest (item 13) scores, as well as the limited range of possible response ratings (i.e., restricted range of 1–5). This was analyzed by a paired *t* test to determine statistically significant differences, if applicable. The open-ended section also invited qualitative reactions or feedback for this specific case.

## Results

The case was run for eight separate sessions with a total of 22 participants, including 17 pediatrics residents, two family medicine residents, two triple-board (pediatrics, child and adolescent psychiatry, and general psychiatry) residents, and one medicine-pediatrics resident. The majority were first-year residents (13), with some second-year and third-year residents (eight and one, respectively). Most residents (86%) had managed an acutely agitated patient in the past, with only three residents without this prior exposure. On different instances, five different facilitators led the case, including four senior residents and one PEM attending, while four different psychiatry content experts assisted the debriefing on different instances of the case. The same SP was used for all sessions, and two sessions were run on any particular day. All participants agreed or strongly agreed that the simulation case was relevant to their work and was realistic (Table 1). Additionally, all but one of the participants agreed or strongly agreed that the debrief created a safe environment and promoted reflection and team discussion (Table 1).

**Table 1.**
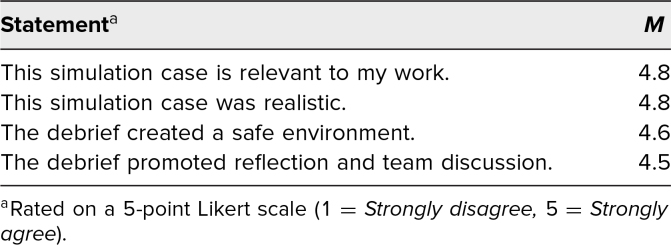
Simulation Survey Results (*N* = 22)

**Figure 1.**
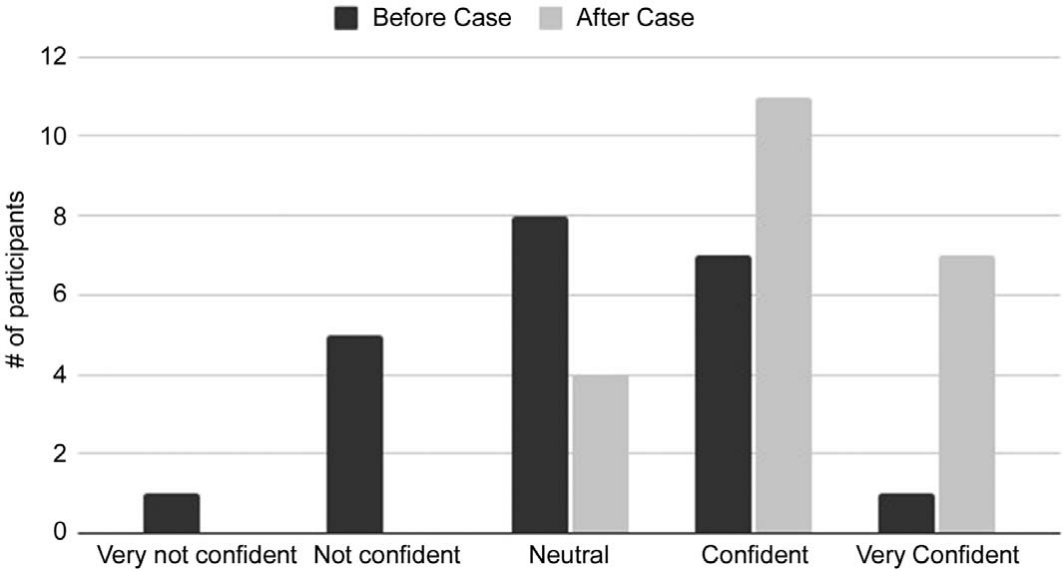
Change in perceived resident confidence in management of acute pediatric agitation after the standardized patient case (*N* = 22). Mean resident confidence prior to the case = 3.1; mean after the case = 4.1 (*p* < .001). Numerical values assigned based on a 5-point Likert scale (1 = *Very not confident,* 5 = *Very confident*).

**Figure 2.**
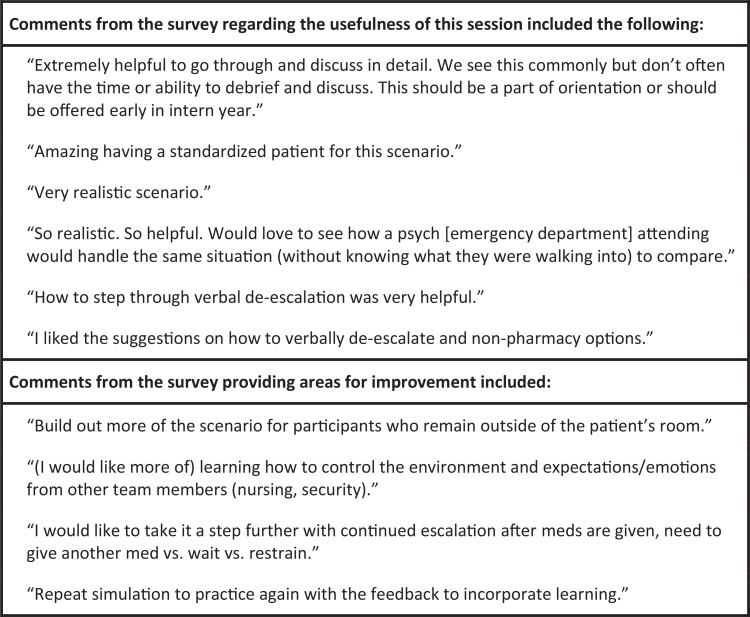
Qualitative survey comments.

As an aggregate, participant confidence in the management of acute pediatric agitation significantly increased after completion of this educational session (*p* < .001; Figure 1). After the session, 18 of 22 participants (82%) rated themselves as confident or very confident in their management skills for acute pediatric agitation, compared to eight of 22 (36%) before the case. Nineteen of 22 (86%) agreed or strongly agreed that the case helped improve their confidence in managing acute agitation scenarios on the inpatient wards. After the case, residents' confidence scores were high for all learning objectives, with mean confidence scores of 4.1 on a 5-point scale (Table 2). Open-ended questions yielded both positive and constructive feedback from learners, and quotations are summarized in Figure 2.

**Table 2.**
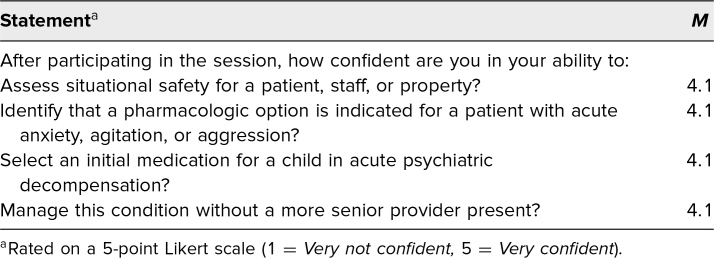
Self-Reported Confidence After Completion of the Case (*N* = 22)

## Discussion

This SP case was created to address gaps in knowledge and skills to manage acute agitation in pediatric behavioral health patients in the inpatient setting. The COVID-19 pandemic has caused an unprecedented increase in mental health diagnoses among children and adolescents in the United States, outpacing the supply of psychiatric resources and the training of pediatric residents.^1,8^ As our results support, residents are not confident in managing pediatric patients with acute agitation. To our knowledge, ours is the first simulated case in the literature that teaches pediatric providers the skills of de-escalation with a psychiatric exacerbation being the primary etiology, rather than secondary to a medical cause such as serotonin syndrome.^19^ The learners appreciated being immersed in this realistic scenario and found this challenging case valuable to their education and training.

This SP case can be adapted in a variety of ways to meet the educational needs of learners. While it was implemented for residents rotating on the pediatric wards, other appropriate learners include PEM fellows, emergency medicine residents, or emergency medicine attendings who may be inexperienced in managing this type of scenario in youth. Similarly, the case can be edited for the developmental level of a medical student by focusing more on verbal de-escalation skills, when to ask for additional support, and ensuring one's own safety in a patient interaction.

In the original iteration of the case, the focus was on improving learners' confidence in these skills; Appendix D can be used to quantify content and skill acquisition over time rather than focus on learners' self-efficacy. The limited materials and personnel needed to implement the case allow it to be generalizable to several types of settings. The case can be utilized in a variety of learning settings, from large academic centers with simulation centers to community-based clinical settings with an empty conference or exam room. Rather than emphasizing the management of the patient, the session can focus on interdisciplinary cooperation and team communication between providers, nursing, and security. We opted not to make teamwork or communication overt objectives because (1) they were not measured in our pre/post surveys and (2) teamwork and communication are inherent in any acute clinical scenario.

We received feedback from some residents that they would have valued this experience earlier in their training, such as during intern orientation. Seeing the scenario presented in this case for the first time in a controlled, safe environment may be particularly helpful for more inexperienced learners. We did not edit the case based on learners' comments as we wanted to ensure the changes were based on more than one individual learner's experience. Our plan was to review comments on a biannual basis to consider adjustments.

Limitations to the implementation of this case must also be acknowledged. First, our sample size was smaller than initially anticipated due to the challenges of scheduling, personnel, and competition with other educational activities. Because of time constraints, we were able to run this activity only twice per month, so not all learners could serve as the active participants; therefore, observers were included and participated in the debriefing portion of the learning activity. The case may be more effectively incorporated into an orientation block or an academic half or full day where more groups could participate. Given the nature of the case, variations in wording used by the SP and facilitators were inevitable despite careful scripting. However, we do not feel that these impacted the case outcome.

There are a few notable biases that may be introduced due to our survey containing retrospective and reactive responses to the topic. Question order bias may exist in participants' pre- and postparticipation confidence responses because they may anticipate their confidence level needing to increase after the intervention of the case. Additionally, the potential for recall bias is present when a retrospective question is asked, and participants' responses to the case may have altered their perceived self-confidence prior to the case's start. However, we felt that the fidelity of the case would be compromised if we administered a presurvey with topic-specific questions. Therefore, we administered surveys only after participation; similarly, objectives for the exercise were not supplied to learners prior to the start of the case.

We were fortunate to have a professionally trained SP with prior experience involving mental health simulated cases. He was a young-appearing adult in his mid-30s, rather than an adolescent SP, recruited primarily due to his schedule's availability, his prior experience with similar cases, and his ability to achieve a degree of escalation that mirrored that of a large adolescent, most important for the fidelity of our case. Although a relatively younger SP is ideal, this may not always be feasible. Likewise, the facilitator playing the role of the nurse must be able to engage learners if they are not actively involved in the verbal de-escalation, so this role is best performed by an experienced facilitator who can provoke discussion.

The physical location of our activity was in a multipurpose room set up to appear like an inpatient room, which affected the fidelity of the scenario. This setting was necessary for several reasons. The SP's interactions were often high volume, occasionally involving inappropriate language and associated loud noises with banging, throwing objects, and so on. This may be disturbing to staff, family, or patients in the surrounding area and should be taken into consideration. These factors prohibited the use of an actual patient room at our institution. We ensured that hospital security was aware of the session to prevent inappropriate activation of the security team. Lastly, learner physical and emotional safety was a top priority. Given the nature of the case, learners could easily be emotionally triggered with defensive and/or stress-response reactions. This further emphasizes the importance of prebriefing the case and debriefing its affective nature by asking learners to reflect on how the case made them feel. We instructed the SP to throw only soft objects (e.g., blanket or pillows) at a wall or the floor, never in the direction of the learners, and to never physically contact the participants; the SP could end the case at any time if feeling emotionally compromised.

We effectively evaluated learner self-efficacy, with residents expressing an aggregate increase in confidence in their own knowledge and abilities. This case had clear objectives, but the intangibility of some objectives (improving interpersonal skills) may have affected postsimulation confidence gain. It is also challenging to measure actual practice change as psychiatric decompensations are unpredictable and vary widely in presentation. As we implement this case further, we will consider ways to longitudinally measure practice change in how residents manage behavioral health emergencies, which may include a proportion of Code Grey cases that result in medication or physical restraint use. Moreover, there is potential for future study of longer-term resident knowledge retention and confidence by administering another posttest several months after the initial case.

Additional future directions include responding to learner feedback by running the case annually to incorporate lessons learned and further develop these skills. Learners also would like to see an example of ideal management by an experienced clinician, which we may incorporate in future debriefings through either a live or a prerecorded case for review by learners. Future studies may further investigate actual provider behavior change in this type of clinical scenario.

As we continue to look for solutions to the current pediatric mental health crisis, it is imperative that curricula teach general pediatric providers how to manage the full spectrum of behavioral health acuity. This SP scenario provides a safe, effective, and realistic scenario for providers to hone these critical skills.

## Appendices


De-escalation Case Facilitator Guide.docxDe-escalation Case Debrief.docxDe-escalation Case Participant Survey.docxDe-escalation Case Critical Action Checklist.docxDe-escalation Case SP Guide.docx
*All appendices are peer reviewed as integral parts of the Original Publication.*
