# Supplementary material for: De-escalation of the Agitated Pediatric Patient: A Standardized Patient Case for Pediatric Residents
Source: MedEdPORTAL. 2024 Mar 8;20:11388. doi: 10.15766/mep_2374-8265.11388 (PMC10920402; doi:10.15766/mep_2374-8265.11388)
Supplement: Supplementary file 1 — De-escalation Case Facilitator Guide.docxDe-escalation Case Debrief.docxDe-escalation Case Participant Survey.docxDe-escalation Case Critical Action Checklist.docxDe-escalation Case SP Guide.docx [file mep_2374-8265.11388-s001.zip › A. De-escalation Case Facilitator Guide.docx]

Appendix A: Standardized Patient Case Facilitator Guide

Date: May 22,2023

Primary Case Author: Adam Kronish, MD

Secondary Case Authors: Charles Wulff, MD, Daniel Alanko, MD, & Robyn Wing, MD, MPH

Standardized Patient Educator: Robyn Wing, MD, MPH

Name of Case: De-escalation of the Agitated Pediatric Patient

Name of Educational Activity: Pediatric Inpatient Teams Educational Session

Use of this appendix: This appendix is to be utilized by facilitators of the standardized patient case to prepare the facilitators and SP for implementation. This activity should last 10-15 minutes in duration, followed by a debrief of the activity, which should take 20-30 minutes as outlined in Appendix B.

Patient Name: Charlie

Chief Complaint: Agitation

Most Likely Diagnosis and Differential With Rationale From History and/or Physical Exam:

Diagnosis: Stress reaction

DDx: Adjustment disorder, psychosis, mania, medication-induced delirium, ADHD

Challenge Question:

Domains: Check all that apply

- Professionalism
- Communication and Interpersonal Skills
- Medical History
- Physical Exam
- Shared Decision-Making
- Patient Education
- Clinical Reasoning
- Documentation
- Handoff
- Presentation
- Other:

Type and Level of Learner: Residents on the pediatric inpatient wards

Case Objectives: Please list specific objectives for each of the domains you have checked above:

By the end of the case,

learners will show increased confidence in being able to:

Communication and Interpersonal Skills/Professionalism:

- 1. Manage an acutely dysregulated patient independently

Medical History/Clinical Reasoning:

1. Assess situational safety for a patient, staff, or property
2. Identify when a pharmacologic option is indicated for patients with acute anxiety, agitation or aggression
3. Select an initial medication for a child in acute psychiatric decompensation based on the individual needs and history of a specific patient

| SETTING: outpatient, in patient, ED, home, nursing home, rehab, group, etc. | Pediatric Inpatient room  An adolescent patient is admitted to the pediatric general medicine service due to emergency psychiatric reasons. They are awaiting placement to an inpatient level of psychiatric care due to lack of bed availability. The learners are called to evaluate the patient for increasing behavioral dysregulation.  On arrival, the patient is angry and borderline combative, but alert and oriented.  Anticipated interventions include utilizing verbal de-escalation techniques and setting up a calm and supportive environment for the patient as first-line interventions, with escalation to voluntary pharmacologic interventions prior to considering medical or physical restraints. |
| --- | --- |
| PATIENT PROFILE: Information about the “patient” that helps select an SP and helps the learner get an understanding of them as a person. SP will know more information about the patient than the learner will ever ask but this allows SP to portray a fully developed patient personality. If none of the items below are particulars for the case, please write “all may be used.” | |
| Age range | 13-17 years old |
| Religious/spiritual background | All may be used |
| Sex (e.g., male, female, intersex, transwoman, transman) | All may be used – male preference |
| Sexual orientation (e.g., heterosexual, lesbian, gay, bisexual, pansexual, queer, asexual) | All may be used |
| Gender expression (e.g., man, woman, genderqueer) | All may be used – masculine-presenting preference |
| Race and ethnicity | All may be used |
| Physical description (e.g., BMI, height range) | All may be used |
| Physical limitations | None |
| Patient appearance (e.g., disheveled, hospital gown, business casual, casual) | Tense-appearing adolescent male standing in corner of room, pacing in the area, alternates between clenching jaw and yelling.  Patient is clearly agitated demonstrating behaviors that may include pacing the room, clenching jaw, tensing shoulders. May be exhibiting signs of psychomotor agitation including trembling hands, fidgeting with fingers or another object, punching a pillow or their own hand. When addressed, answers are initially short 1-5 words, terse and pressured, which may escalate to yelling. Avoids eye contact unless their name is explicitly said to them.    Clothing is hospital provided scrubs for patients admitted medically while awaiting psychiatric placement |
| Moulage + location (e.g., none, bruises, scars, body piercing, tattoos) | None |
| Affect (e.g., pleasant, cooperative) | Agitated dysregulated ; mood is “frustrated” escalating to angry |
| Family group (e.g., who is family, who they live with) | Has at least 1 parent who may be present in the case as a facilitator or SP. Lives in a 2-parent home with one younger sibling (genders of parents and sibling are unimportant to the case) |
| Education | High school education appropriate for age |
| Level of health literacy | Appropriate for age but low |
| Employment, if any - present and past, noting any current stresses | No employment ever |
| Home/homeless - type of dwelling, number of stories, owned or rented | Lives in a suburban home owned by parents |
| Financial situation - any current stresses | Lower middle-class family, no recent financial stressors |
| Insurance status (e.g., un/under/insured, public/private, HMO/PPO) | Private Insurance |
| Habits (i.e., diet, exercise, caffeine, smoking, alcohol, drugs) | - Typical American diet - 0-2 caffeinated beverages daily - No regular exercise - Occasional marijuana use - Several times a week currently; last use yesterday |
| Activities (i.e., hobbies, sports, clubs, friends) | - He loves playing video games and looking up videos online.  - Has few friends at school, but several “online” friends  - He likes music and that calms him, as does doing artwork. |
| Typical day - what is the usual daily routine | 6:30AM – Woken up by parent for school  7:15AM – Bus to school  7:50AM to 2:15PM – School  2:30 PM to 3:10PM – Bus to home  3:10PM to 10PM – Video games, listening to music, homework, dinner  10PM to midnight - Bedtime |

| CASE INFORMATION | |
| --- | --- |
| Stage Setting by the Facilitators: | The learner is working as the covering overnight resident for the general pediatric floor. A “Code Gray” (Behavioral Health Emergency Code) was just called for a new patient that arrived on the floor after boarding in the emergency room for the past 24 hours. The learner’s team received a limited handoff of the patient, specifically told by facilitator to students; for example: “this is a 15-year-old male with a history of PTSD and trauma admitted for depression, now awaiting inpatient psych placement.”    At time of entry into the room, the patient is standing in the corner of the room and yelling. |
| Chief Concern: What the patient will say when greeted by the student. The patient’s primary reason for seeking medical care often stated in their own words. | “No one is listening to me. Let me out of here!” |
| Additional Concerns: Other, if any, concerns the patient has today (i.e., symptoms, requests, expectations, etc.) that will become part of set agenda. | None |
| THE PATIENT’S STORY: The SP will be asked to tell their symptom story and the personal and emotion impact for each of their concerns. You will want to write this in the patient’s voice. The symptom story should be able to answer this question: “Tell me more about [chief concern/additional concern], starting at the beginning and bringing me up to now.”  The personal context should be able to answer questions concerning the broader personal/psychosocial context of symptoms, especially the patient’s beliefs/attributions.  The emotional context should be able to ask how are you doing with this, how does this make you feel, how has this affected you emotionally? IMPACT: How has this affected your life? How has this been for your family? | SP offers no additional history  Bedside RN offers: *“Charlie just got settled in his room and asked if he could play some video games to pass the time. When I told him we had none available, he got very upset and starting yelling, throwing his pillows and sheets, and cursing. I tried to get him to calm down but he is inconsolable.”*    Parent (If a role is present; otherwise bedside RN may add) offers: “*At home he would frequently go from 0 to 100 like this in response to small changes. He was admitted to the hospital last year for similar problems and was in a good place mentally up until a few weeks ago.”* |
| HISTORY OF PRESENT ILLNESS: Although some of the HPI will be given in the patient’s symptom story, the learners will expand the story during the direct question section. Below, describe the detailed history, usually about the chief concern, which the student must develop in order to make a useful assessment of the problem: | |
| Onset (when; gradual or sudden) & Setting (what was going on or where was patient when symptoms first noticed?) | Onset was sudden within 20-30 min of arrival to inpatient room |
| Duration (how long) | Has been yelling for the past 10 minutes |
| Time relationships (frequency, constant or intermittent) | N/A |
| Location | N/A |
| Radiation | N/A |
| Quality | N/A |
| Amount | N/A |
| Aggravated by what | In the past, symptoms have worsened with intramuscular diphenhydramine (Benadryl shot) |
| Relieved by what | Can distract himself at home with music, video games or art |
| Associated with what | N/A |
| Attitude (what does the patient think is the problem, and how do they feel about it) | Patient “is tired of waiting” and wants to “go home”. Wants to not be as reactive but his “mind is racing”. |
| Overall course | Overall – patient presented 1-2 days ago to ED for suicidal ideation, was deemed at risk of harm to self and signed voluntary consent for inpatient psychiatric treatment. He waited in the ER for a full 24 hours after this decision was made and then transferred to an inpatient room. Within 30 min of arrival to inpatient room, patient began yelling and pacing the room. |
| REVIEW OF SYSTEMS: Significant positives and negatives | |
|  | + heart racing, racing thoughts, inability to stay still  ROS in all other systems is otherwise negative |
|  |  |
|  |  |
|  |  |
|  |  |
| Past medical history | -  He has a history of depression and PTSD.  - Has intermittent asthma, well-controlled  -  He has a therapist who he sees every 2 weeks and a psychiatrist whom he last saw 2 months ago.  -  He has a history of trauma related to physical arm restraints in the past. |
| Medication allergies (name and reaction) | NKDA  Adverse reaction: Paradoxical reaction to diphenhydramine. “He had a very bad reaction specifically when he got the Benadryl shot, where he ramped up rather than calmed down.” |
| Environmental allergies (name and reaction) | None |
| Illnesses | None |
| Vaccinations | Up to date |
| Surgeries | None |
| Accidents/injuries/trauma | None |
| Hospitalization | None |
|  | |
| Inclusive sexual and reproductive history | |
| Sexual practices  Sexual partners  Protection: Use of safer sex practices  Use of birth control if appropriate  Risk of intimate partner violence | No prior sexual activity |
| OB/GYN history | Age of onset of menses - N/A for male; 13 for female  Age of menopause - N/A  Number of pregnancies - 0  Number of live births - 0  Number of miscarriages - 0  Number of abortions - 0 |
| Medications | - Fluoxetine 20mg in AM for depression - Albuterol PRN for intermittent asthma |
| Tobacco products:   - Cigarettes - Cigar - Pipe - Chew - E-cigarettes | X Never   - Past - year started/year quit - Current   - Quantity   - # of years |
| Alcohol   - Beer - Wine - Liquor - Other | X Never   - Past - year started/year quit - Current   - Quantity   - # of years |
| Drugs  X Marijuana/Cannabis/Weed   - Cocaine - Heroin - Meth - IV - Inhalants - Other | - Never - Past - year started/year quit   X Current   - - Quantity – Several times a week currently; typical is once/month   - # of years – About a year |
| Diet (describe) | Typical “teenage” diet – enjoys fast food, skips breakfast, very little fruits/veggies |
| Exercise (describe) | Gym class |
| List any other important social history or information important to this case | None |
| Family history |  |
| Mother, father, siblings, grandparents, and other significant findings | - Father with bipolar disorder and opioid use disorder - Sister with asthma depression and past suicide attempts |
|  |  |
| Physical Exam - List exam maneuvers expected for this case and any abnormal findings that SP will simulate. (tenderness, hyper-hypo reflex, rebound, weakness, etc.)   \| Physical Examination \| \| \| --- \| --- \| \| General \| Tense-appearing adolescent male standing in corner of room, pacing in the area, alternates between clenching jaw and yelling. Mood is “frustrated” \| \| HEENT \| Moist mucous membranes, no conjunctival injection \| \| Lungs \| Patient does not allow examiner to auscultate; breathing quickly but comfortably \| \| Cardiovascular \| Patient does not allow examiner to examine \| \| Abdomen \| Patient does not allow examiner to examine \| \| Neurological \| Moves all extremities equally bilaterally, no dysarthria, normal extraocular movements \| \| Skin \| Linear scars in different stages of healing on bilateral forearms (if asked explicitly about this part of the exam), no active bleeding \| \| GU \| Patient does not allow examiner to examine \| \| Psychiatric \| Mood: “frustrated”  Alert and appears stated age, oriented to person, place and time, normal speech and language, irritable affect with congruent mood, linear thoughts.  Endorses passive suicidal ideation without a specific plan or intent at this moment \| | |
| PHYSICAL EXAM FINDINGS |  |
| 1. Written in layperson’s terms | Tense and angry adolescent who is clenching jaw, holding tight fists, walking around the room and unable to sit still. |
| 1. General appearance - affect, appearance, position of patient at opening (i.e., sitting, lying down, holding abdomen, etc.) | Tense-appearing adolescent male standing in corner of room, pacing in the area, alternates between clenching jaw and yelling.  Mood is “frustrated” |
| 1. Vital signs | T: 37.0 C, HR 97, RR 14, BP 120/70, SpO2 100%, Weight 60 kg |
| 1. Specific findings and affect | Affect: agitated |
| 1. Response to certain physical movements | Becomes more angry if providers physically approach him without building a rapport  Does not allow provider to physically examine him closely (no heart, lung, abdominal exam) |
| DIAGNOSIS AND DIFFERENTIAL |  |
| Diagnosis with support from positive and negative history and PE findings | 1. Acute stress reaction – supported by timing of behavior response with transition (from ER to inpatient floor), baseline anxiety/depression, logical reasoning, acuity (adjustment disorder usually persists for >3-6 months of a stressor) |
| Differential with support from positive and negative history and PE findings | 1. Acute mania – supported by +Fhx of bipolar disorder, psychomotor agitation present, speech is loud and rapid; against this Dx is no flight of ideas/tangential thinking, no insomnia, is not easily distractible, no grandiosity, no prior history of mania or manic symptoms 2. Acute Psychosis – supported by psychomotor agitation and history of cannabis use; against this Dx is a lack of prior history of psychosis, no delusions, not appearing to respond to and does not endorse internal auditory of visual stimuli/hallucinations 3. Medication/Substance Reaction – supported by history of adverse reaction; against this Dx is lack of medication in the past 12 hours and has been taking daily medicines as prescribed 4. ADHD- supported by inability to stay still and hyperactivity; against this Dx is lack of prior history, acuity of the scenario (ADHD would be more chronic) |
|  |  |
| MANAGEMENT OR DIAGNOSTIC PLAN | See Instructor Notes below. Overview:   1. Verbal de-escalation techniques are attempted by a group leader 2. Team becomes aware of prior history of paradoxical reaction to diphenhydramine 3. Oral alternative to diphenhydramine is offered and accepted by patient and he de-escalates |
|  |  |
| PROFESSIONALISM ISSUES OR CHALLENGES | See “Anticipated Management Mistakes” below. |

| Instructor Notes - Changes and CASE Branch Points | | |
| --- | --- | --- |
| Intervention / Time point | Change in Case | Additional Information |
| At onset of case, one learner should identify themselves as leader of the situation |  | If not done, bedside RN should inquire, *“who is in charge?”* |
| *If parent is present,* should ask what is helpful for calming patient down | Parent says that Ativan was tried in the emergency department yesterday and that made him nice and sleepy. At home, he stomps around his room and plays music. |  |
| *At 3 min* | If no one has directly engaged with the patient, he will become more disruptive and say “*No one is listening to me”* |  |
| *At 5 min* | if not yet asked, parent or bedside RN offers that patient responds well to deep breathing and/or music in the past. |  |
| Engages with patient to identify stressor | Patient makes eye contact with provider and expresses feeling frustrated and wishes to leave the hospital. | If not done, patient closes eyes and stomps on ground or kicks wall/chair saying *“Let me out of this place”.* |
| Utilizes 1 de-escalation technique with patient:  ● listen + validate distress  ● offer distraction/calming activity  ● offer patient a choice  ● remove dangerous objects (such as a plastic knife, IV pole, pen, etc.)  ● remove sources of stress (such as a phone with hurtful text message or a non-supportive/argumentative parent) | Patient makes eye contact with provider. Will endorse that they appreciate support, but feel their mind is racing and they are having trouble calming down. | If not done, patient closes eyes and stomps on ground or throws pillow/blanket yelling *“Let me out of this place”.* |
| If asks for notes from chart on past admission | Nurse will summarize notes: “*Patient is taking Prozac 20mg daily, in addition to albuterol PRN. Chart notes prior history of receiving Benadryl with paradoxical reaction.”* | History of paradoxical/adverse reaction to Benadryl identified, so this medication is *NOT* offered |
| Provider offers patient oral lorazepam (Ativan) or other non-Benadryl PO agent to stay safe | Patient accepts medicine and takes tablet. | If not done, patient continues to escalate. |
| If learner seems unsure about a specific medication that is not Benadryl | Bedside nurse asks*, “Would lorazepam could work for this patient?”* |  |
| If Benadryl is given to patient at any point | Patient closes eyes and stomps on ground or kicks wall/chair saying *“Let me out of this place”.* |  |
| Once any medicine is given | Patient takes medicine and bedside RN will ask, *“how long before we can try something else?”* | Learner answers: 20-40 minutes for onset    If not answered correctly, bedside RN will ask if we can give another dose now since the patient is still “worked up”. |
| If physical restraint or IM medication is offered at any point by learner | Patient escalates further due to prior trauma from physical restraints, and bedside RN calls security and case ends. |  |
| Before case ends and while the patient is not calm, bedside RN or other team member asks “*Can’t we just put him in the soft restraints? He’s being really disruptive to the other patients.”* | Either support or leader MD can decline this request, citing either trauma history and/or that only the least restrictive interventions should be used. |  |

Ideal Scenario Flow

*The learners arrive at the bedside and find a bedside nurse (and parent, if able to have 2 facilitators) at the doorway and a teenager standing in the corner of a room with arms crossed and pacing the room. They ask for a brief report of the events leading up to the Code Gray/Behavioral Code and are provided the “offered’ information above. Vital signs are provided and one learner approaches the patient while others talk with the nurse and parent. The patient is initially still angry and escalates with raised voice until addressed directly by the learner. When asked, the patient says he is frustrated by continuing to be in the hospital for so long. Through either patient or parent interview, the learners discover helpful calming techniques in the past that have included deep breathing and music for this patient. They also learn that he has had a paradoxical reaction to Benadryl as well as a traumatic experience with physical restraints. They engage in verbal de-escalation and the patient says that he still feels like his mind is racing and is having trouble staying calm. The learners offer the patient oral lorazepam or another acceptable oral agent that is not Benadryl. The patient agrees to take the medicine, and the team agrees to re-evaluate in at least 20 minutes for therapeutic effect. The learners explicitly do not offer to give the patient intramuscular medicine or physical restraints in part due to trauma history and also to use least restrictive interventions. Case ends soon after oral medication administered and proper next steps are discussed.*

Anticipated Management Mistakes

Specific prompts and responses for anticipated management mistakes are outlined in the “Instructor notes- changes and branch points” section above. To summarize, they include:

1. *Not directly engaging with the patient: There may be a delay in talking directly to the patient to de-escalate in part due to anxiety about engaging in the dysregulated patient. Some learners may focus more on obtaining history from parent/other providers.*
2. *Lack of knowledge of medication options for agitation: General pediatric trainees may not be familiar with many medications other than diphenhydramine (Benadryl). In this case, it is most important to recognize that patients with psychiatric histories may have experiences in the past that are guiding for future encounters. Facilitators may offer at least 1 option if it seems this is a struggle.*
3. *Offering patient diphenhydramine (Benadryl): If the patient is offered diphenhydramine first and it is administered, the patient’s agitation continues to escalate further with even more barrier to distractibility. A second-line agent may be offered by the bedside RN (such as lorazepam) after administration of diphenhydramine; alternatively, prior to the administration of diphenhydramine, the parent or bedside RN may note a history of an adverse reaction to Benadryl in the past.*
4. *Attempting to place patient in physical restraints: If the team opts to place the patient in restraints at any point, the patient will escalate further while security is called by the bedside RN. The case ends immediately and the debrief will focus around indications for physical restraint, including imminent harm to self or others. Facilitators will highlight that leaving the room and allowing another provider to attempt de-escalation or calling for additional support would be more appropriate in this case as threats to self/others/property did not occur.*
5. *Call security immediately at start of case: Trainees may be accustomed to security and/or other support staff being called concurrently for behavioral health emergencies. If security is called immediately, the bedside RN may call for security but have the team continue to attempt de-escalation while “security is on the way”. The bedside RN may then say that security is “outside the room awaiting instructions”, at which point the learners will be given another chance to decide if security in the room would be helpful or hindering to the de-escalation of the agitated patient. If security is offered to come into the room ,the case will end immediately and a discussion of indications/contraindications to additional support by security staff will be highlighted in the debrief.*

*A consult to psychiatry is called for additional support before engaging with patient:*  *It would not be incorrect to have the team simultaneously consult psychiatry for a stepwise plan for PRN medications for agitation. Failure to engage with the patient first however would be a mistake. As with any consultation to a specialist, a thorough understanding of the presentation, exacerbating factors/triggers to the event, and a specific consult question; none of this information is available without first engaging with the patient. If this is attempted, the learners will be informed that psychiatry is not in-house at this time but have been paged. Psychiatry “recommendations” of PRN medications may be given only if verbal de-escalation is first attempted. The “psychiatrist” (may be acted via phone or relayed by the bedside RN) may prompt the learners to select a medication before giving recommendations for/against any particular agent*
