# Supplementary material for: De-escalation of the Agitated Pediatric Patient: A Standardized Patient Case for Pediatric Residents
Source: MedEdPORTAL. 2024 Mar 8;20:11388. doi: 10.15766/mep_2374-8265.11388 (PMC10920402; doi:10.15766/mep_2374-8265.11388)
Supplement: Supplementary file 1 — De-escalation Case Facilitator Guide.docxDe-escalation Case Debrief.docxDe-escalation Case Participant Survey.docxDe-escalation Case Critical Action Checklist.docxDe-escalation Case SP Guide.docx [file mep_2374-8265.11388-s001.zip › B. De-escalation Case Debrief.docx]

**Appendix B. De-escalation Case Debriefing Guide**

Debriefing, an interactive, bidirectional and reflective discussion, is the most important feature of simulation and standardized-patient based education.^^[[1]](#footnote-1)^^ There are several methods used to conduct debriefing in healthcare simulation and any would provide an adequate educational reflection for the brief scenario in our session. The key to all debriefing methods is to provide a supportive environment to ensure psychological safety, use open-ended questions to encourage active participation, and make sure to address the learning objectives. The debriefing method outlined below, modeled after PEARLS^^[[2]](#footnote-2)^^, provides a simple yet complete structure for efficient debriefing.

Each debriefing session starts with a brief “Introduction” or “Pre-brief” during which the ground rules for the debriefing session are set for all participants. This encourages a respectful and safe learning environment for optimal education and participation. The “Reaction” phase of debriefing aims to encourage learners to process their emotions surrounding the case itself and/or their individual performance in the case. This is done early in the debrief in the hopes of allowing learners to move past these emotions into the remainder of the debrief. The “Description” phase aims to clarify the facts of the case to develop a shared understanding of the case. The “Analysis” phase explores a variety of performance domains, including teamwork and communication, medical decision making and technical skills; this section is meant in particular to address the learning objectives outlined below. Facilitators use this portion of the debrief to ensure that specific learning objectives are addressed. Lastly, the “Summary” phase encourages learners to highlight their take home points and gives facilitators a final opportunity to ensure that learning objectives have been covered.

Learning Objectives:

To increase learners’ confidence in the abilities to:

1. Assess situational safety for a patient, staff or property; this includes recognizing the role of the environment in helping/hindering de-escalation
2. Manage and attempt to verbally de-escalate an acutely dysregulated patient independently
3. Identify when a pharmacologic option is indicated for patients with acute anxiety, agitation or aggression
4. Select an initial medication for a child in acute psychiatric decompensation based on the individual needs and history of a specific patient

| Debriefing Guide with PEARLS^2^ approach: | |  |
| --- | --- | --- |
| **Debriefing Phase** | Suggested questions/phrases | Case-Specific Discussion Points & Reference Materials |
| **Introduction/Pre-brief** | We are now going to take the next 15-20 minutes to debrief. This part of our session is actually more important than the actual case. As a reminder, our ground rules are as follows:   - 1. This is meant to be a safe learning environment   2. Simulation/Standardized Patient activities create a formative, interdisciplinary learning environment - no one is being evaluated (except the instructors because we constantly evaluate ourselves on how to provide better educational sessions).   3. We all agree to the basic assumption that everyone here is intelligent, well trained, and cares about doing their best.   4. Thus, we all agree to the highest standard of professional conduct and courtesy to our colleagues in this debrief and in any further discussions about this mock code. |  |
| **Reactions** | - That was a challenging/stressful/difficult case. How are you feeling? Any initial reactions? - What part of this scenario was particularly challenging? Why? | - Validate learner’s reactions and emotions - Invite thoughts from several learners |
| **Description** | - Can someone please summarize the case for us? - Does anyone have anything to add about the facts of the case? |  |
| **Analysis** ^^[[3]](#footnote-3)^^ | **Assess situational safety for a patient, staff or property** | |
|  | - “I noticed you *[quickly/took a while to]* have the patient sit or lower their voice and assess for any threatening objects in the room. This was [*great/could be problematic]* because in assessing a behavioral emergency, safety for the patient *and* staff are the first priority.”   **Recognize the importance of environmental factors that may help or hinder de-escalation**   - “I noticed you [*quickly/took a while]* to change the environment around this patient. This was [*great/could have been problematic]* since the differences in the environment and setting may exacerbate or relieve an escalating patient.” | - Assessing for safety includes checking for objects that may allow injury to self or others (ex: sharp objects, utensils, drawing instruments, strings/bands, IV poles, etc.) - If the patient is a threat to themselves or others and verbal de-escalation fails, this would be an indication for an as-needed medication for agitation, or potentially physical restraint. - Environmental factors may contribute to an escalating patient: - closed or aggressive body language - loud voice - multiple people staring - security at the doorway - not being at the same level as the patient, etc. |
|  | **Manage and attempt to verbally de-escalate an acutely dysregulated patient without a more senior provider present** | |
|  | - “I noticed you *[quickly/took a while]* attempted to engage the patient verbally before reaching for medication/physical restraint. This was [*great/could be problematic]* since utilizing verbal de-escalation strategies alone may be sufficient. - “What [*helped/hindered*] you to [*recognize/treat*] it?” | Verbal De-Escalation skills may include:^^[[4]](#footnote-4)^,^[[5]](#footnote-5)^^   - Provide support and redirect in a calm neutral tone (“I see you’re feeling ____/want ____, I want to help”) - Use short sentences and simple language - Use non-threatening body language (unclenched hands, calm expression, maintain some distance). Try to appear calm even if you are not. - Assess sensory triggers/overstimulation (excess noise, bright lights, etc) - Offer sensory tools (play dough, fidgets), distractions (drawing, ipad), or other “acts of kindness” (blanket, snacks) - Offer coping strategies (deep breathing, yoga stretches, physical and tactile stimulation) - Recognize that dysregulation is often secondary to a patient’s trauma history. Therefore, trauma-focused care and communication are vital. Consider what has happened to the patient that could lead to this situation (i.e., patients may react differently to male providers, people touching them, etc) |
|  | **Identify that a pharmacologic option is indicated for patients with acute anxiety, agitation or aggression** | |
|  | **Select an initial medication for a child in acute psychiatric decompensation** | |
|  | - “I noticed you *[quickly/took a while]* to [*initiate/identify*] an appropriate oral pharmacologic treatment for agitation. This was *[great/could have been even better]* because verbal de-escalation may be insufficient and oral medications taken willingly prevent complications secondary to restraint. - “What [*helped/hindered*] you to [*recognize/treat*] appropriately? | Indications for as-needed medication for agitation (by mouth or intramuscular): Patient poses risk to themselves, to staff/family, or to hospital property and verbal strategies are inappropriate or ineffective  Notes:   - Patients should be involved in medication choice when possible - Offer in a comforting manner along with other non-pharmacologic interventions   Possible oral medications may include (onset of action 15-30 min):^^[[6]](#footnote-6)^^   - Hydroxyzine (Atarax) - Diphenhydramine (Benadryl) - Lorazepam (Ativan) - Olanzapine (Zyprexa) - Quetiapine (Seroquel) - Risperidone (Risperdal) - Aripiprazole (Abilify) - Less commonly used antipsychotic agents: Chlorpromazine (Thorazine), Ziprasidone (Geodon), Haloperidol (Haldol),   Most of the above are available in IM form (onset of action 5-10 min)  Be aware of over-sedation (from excessive and/or frequent dosing):   - Diphenhydramine/Hydroxyzine: anticholinergic effects, prolonged QT, paradoxical reactions - Benzodiazepines: paradoxical agitation, respiratory depression - Antipsychotics: anticholinergic effects, prolonged QT, decreased seizure threshold, dystonic reactions, neuroleptic malignant syndrome |
| **Teamwork & Communication** | **Identifying Team Roles and Responsibilities**   - “From my perspective, it looked like you (*did/did not)* have clear team roles. I think this is (*great/concerning)* because clear team roles can help a team function smoothly, improving how quickly interventions take place and reducing errors.” | Team leader   - Clear direction, coordination, timely interventions - Stands away from patient to maintain safety and to see the big picture |
|  | **Forming a Pre-brief or Huddle**   - “I noticed that your team *(did/didn’t/took a while to)* (*brief* *prior to the initial patient assessment/huddle after the initial evaluation).* I thought this (*was* *great/could have helped to*) facilitate patient care.” - “What *(helped/hindered)* your team from (*briefing/huddling*)?” - “How did that impact your team?” - “What could your team have done differently?” | The goal of a brief/huddle is to create a shared mental model. Assure all team members know what the working diagnosis is, management priorities and next steps in care.   - Everyone on the team is responsible for making this happen. Anyone can ask for a brief/huddle. Brief/huddle is usually led by the team leader. - If one team member doesn’t have situational awareness or know the plan, s/he is probably not alone. |
|  | **Utilizing Directed Call-Out**   - “I noticed that you (*did/didn’t/intermittently*) used (*team member’s names/roles/eye contact*) when (*calling out orders/asking for assistance*). I thought this was (*great/could have been more directed*) in order to facilitate communication.” - “What did you notice about orders/questions that were asked?” - “How did this impact your team?” | Directed call out is a tactical communication skill to assure that important orders/questions are specifically directed to one individual (rather than called out into the air).  Example:   - “Jennifer, please place an order for 1 mg of oral Ativan.” |
|  | **Using Closed-Communication/Check-Back**   - “I noticed that you used closed-loop communication *(consistently/rarely)*. Closed-loop communication can be critical for catching errors and assuring that *(information/an order/a request)* is heard.” - “Tell me about your communication loops” - “How did that impact your team?” - “Has anyone seen problems with this in a patient de-escalation? Can you tell us about that?” - “Has anyone seen closed loop communication prevent an error?” - “How could you do it differently next time?” | Closed loop communication/check back is a strategy that requires verification of information. This enables the sender of the message to verify it has been heard and heard correctly. It enables the receiver to confirm what they heard is correct.  Examples:   - Team leader “Float nurse, please offer the 1 mg of oral Ativan” - Float nurse: “1 mg of oral lorazepam given.” - Team leader “Thank you” |
| **Summary** | - What unanswered questions do you have about this case? - What is your take home point? (go around the room and have every learner state one) |  |

**De-Escalation Debriefing Checklist**

This checklist is a shortened version of the above to be used by seasoned debriefers who do not need all the above prompts and information.

|  | **Debriefing Topic** | **Example Prompt(s)** | **Completed?** |
| --- | --- | --- | --- |
| **1** | Introduction/Pre-Briefing | See script in table above |  |
| **2** | General reactions to the case | “That was a challenging/stressful/difficult case. How are you feeling? Any initial reactions?” |  |
| **3** | Description | “Can someone please summarize the case for us?” |  |
| **4** | Assess the safety of the situation for patient and staff | “How did you assess the safety of the situation?” |  |
| **5** | Evaluate environmental factors on de-escalation in relation to safety for patient, staff, & property | “What in the environment was helpful for de-escalating this patient and what could have been changed?” |  |
| **6** | Verbal De-Escalation Skills | “How did you approach verbally de-escalating the patient?” |  |
| **7** | Indications for PRN medication | “Why did you (or didn’t you) decide to give the patient medication?” |  |
| **8** | Medication choice | “What are the options for the type and route of medication administration?”  “How did you choose ‘X medication’ to offer this patient?” |  |
| **9** | Indications Intramuscular medication and/or restraint | “What would the indications have been to physically restrain or non-consensually administer IM medication to the patient?” |  |
| **10** | Teamwork & Communication | “Talk about how you worked as a team, how you decided roles, and how you felt communication amongst the team members went.” |  |
| **11** | Summary | “What unanswered questions do you have about this case?”  “What is your take home point?” (Go around the room and have every learner state one) |  |

1. Sawyer T, Eppich W, Brett-Fleegler M, Grant V, Cheng A. More Than One Way to Debrief: A Critical Review of Healthcare Simulation Debriefing Methods. *Simul Healthc*. 2016;11(3):209-217. [↑](#footnote-ref-1)
2. Eppich W and Cheng A. Promoting Excellence and Reflective Learning in Simulation (PEARLS): Development and Rationale for a Blended Approach to Health Care Simulation Debriefing. Sim in Healthcare. 2015:10 (2): 106-115. [↑](#footnote-ref-2)
3. ^Adapted from format of Appendix F from the following citation: Shubin, C., Iyer, S., Pearce, J. et al. “Pediatric Emergency Medicine Didactics and Simulation (PEMDAS): Serotonin Syndrome.”^ *^MedEdPORTAL^*^, 2020, https://doi.org/10.15766/mep_2374-8265.10928.^  [↑](#footnote-ref-3)
4. ^Richmond, Janet, et al. “Verbal De-Escalation of the Agitated Patient: Consensus Statement of the American Association for Emergency Psychiatry Project Beta De-Escalation Workgroup.”^ *^Western Journal of Emergency Medicine^*^, vol. 13, no. 1, 2012, pp. 17–25., https://doi.org/10.5811/westjem.2011.9.6864.^  [↑](#footnote-ref-4)
5. ^Raypole, Crystal. “Grounding Techniques: Exercises for Anxiety, PTSD, & More.”^ *^Healthline^*^, Healthline Media, 13 June 2022, https://www.healthline.com/health/grounding-techniques#physical-techniques.^  [↑](#footnote-ref-5)
6. ^Chun, Thomas H., et al. “Evaluation and Management of Children and Adolescents with Acute Mental Health or Behavioral Problems. Part I: Common Clinical Challenges of Patients with Mental Health and/or Behavioral Emergencies.”^ *^Pediatrics^*^, vol. 138, no. 3, 2016, https://doi.org/10.1542/peds.2016-1570.^  [↑](#footnote-ref-6)
