# Supplementary material for: De-escalation of the Agitated Pediatric Patient: A Standardized Patient Case for Pediatric Residents
Source: MedEdPORTAL. 2024 Mar 8;20:11388. doi: 10.15766/mep_2374-8265.11388 (PMC10920402; doi:10.15766/mep_2374-8265.11388)
Supplement: Supplementary file 1 — De-escalation Case Facilitator Guide.docxDe-escalation Case Debrief.docxDe-escalation Case Participant Survey.docxDe-escalation Case Critical Action Checklist.docxDe-escalation Case SP Guide.docx [file mep_2374-8265.11388-s001.zip › C. De-escalation Case Participant Survey.docx]

**Appendix C. De-escalation of the Acutely Agitated Pediatric Patient: Case Survey**

1. What is your current level of training?

☐ PGY 1 ☐ PGY 2 ☐ PGY 3 ☐ PGY 4

☐ PGY 5 ☐ Other ____________

2. What residency program are you in?

☐ Pediatrics ☐ Medicine-Pediatrics ☐ Triple board ☐ Family Medicine

☐ Psychiatry ☐ Other ____________

3. Have you ever had to manage this condition before?

☐ Yes- on the wards ☐ Yes- in the ED ☐ No ☐ Other ______________

4. How confident were you in the management of the acutely agitated pediatric patient prior to participating in this simulated case?

☐ Very not confident

☐ Not confident

☐ Neutral

☐ Confident

☐ Very confident

**Please rate your agreement with the following statements:**

|  |  | Strongly Disagree | Disagree | Neither Agree nor Disagree | Agree | Strongly Agree |
| --- | --- | --- | --- | --- | --- | --- |
| 5 | This simulation case provided is relative to my work. |  |  |  |  |  |
| 6 | This simulation case was realistic. |  |  |  |  |  |
| 7 | This simulation case improved my confidence in managing acute agitation scenarios on the inpatient wards. |  |  |  |  |  |
| 8 | The debrief created a safe environment. |  |  |  |  |  |
| 9 | The debrief promoted reflection and team discussion. |  |  |  |  |  |

**After participating in this session, how confident are you in your ability to:**

|  |  | Very Not confident | Not confident | Neutral | Confident | Very Confident |
| --- | --- | --- | --- | --- | --- | --- |
| 10 | Assess the safety of a situation for a patient, staff, family, and property. | □ | □ | □ | □ | □ |
| 11 | Identify when a pharmacologic option is indicated for a patient with acute anxiety, agitation, or aggression. | □ | □ | □ | □ | □ |
| 12 | Select an initial medication for a child in acute psychiatric decompensation. | □ | □ | □ | □ | □ |
| 13 | Manage this condition independently. | □ | □ | □ | □ | □ |

14. What did you take away from this case and/or how will it change your practice?

____________________________________________________________________

____________________________________________________________________

____________________________________________________________________

15. What specific changes would you make to improve this scenario?

____________________________________________________________________

____________________________________________________________________

16. Other comments or suggestions:

____________________________________________________________________

____________________________________________________________________

____________________________________________________________________

**Thank you for taking the time to complete this survey!**
