# Supplementary material for: De-escalation of the Agitated Pediatric Patient: A Standardized Patient Case for Pediatric Residents
Source: MedEdPORTAL. 2024 Mar 8;20:11388. doi: 10.15766/mep_2374-8265.11388 (PMC10920402; doi:10.15766/mep_2374-8265.11388)
Supplement: Supplementary file 1 — De-escalation Case Facilitator Guide.docxDe-escalation Case Debrief.docxDe-escalation Case Participant Survey.docxDe-escalation Case Critical Action Checklist.docxDe-escalation Case SP Guide.docx [file mep_2374-8265.11388-s001.zip › D. De-escalation Case Critical Action CheckList.docx]

**Appendix D: Critical Action Checklist**

Below are listed important steps for providers to consider and perform when treating an escalating patient. Please mark whether these were done during the case or spoken about afterwards in the debrief.

| **Critical Action** | **Performed during case? Y/N** | **Discussed afterwards during debrief? Y/N** |
| --- | --- | --- |
| Introduced self to patient |  |  |
| Assessed room safety including:   - Keeps self between patient and door - Assessed and removed any items in the room that may become dangerous to patient and/or staff |  |  |
| Approached patient in a calm way, examples include:   - Used a non-aggressive stance - Used simple/plain language - Uses validating statements - Leaves space in between patient and provider |  |  |
| Attempted Verbal De-escalation with examples including but not limited to:   - Assessing sensory triggers/overstimulation - Offering distractions such as sensory tools, drawing, iPad, or other “acts of kindness” (blankets, snacks) - Offer coping strategies (deep breathing, yoga stretches, physical and tactile stimulation) |  |  |
| **Medical History** |  |  |
| Enquired about patient’s PMH including:   - Medications patient takes - Allergies to medications |  |  |
| Enquired about paradoxical reactions to medications |  |  |
| Involved patient and/or parent in choice of medication |  |  |
| Confirmed proper medication and dosing with team and nursing staff |  |  |
| **Teamwork and Communication** |  |  |
| Identified a team leader |  |  |
| Utilized closed loop communication when interacting with other team members |  |  |
| Demonstrated proper “crowd control” (asking unnecessary/ancillary staff to leave the room) |  |  |
