# Supplementary material for: De-escalation of the Agitated Pediatric Patient: A Standardized Patient Case for Pediatric Residents
Source: MedEdPORTAL. 2024 Mar 8;20:11388. doi: 10.15766/mep_2374-8265.11388 (PMC10920402; doi:10.15766/mep_2374-8265.11388)
Supplement: Supplementary file 1 — De-escalation Case Facilitator Guide.docxDe-escalation Case Debrief.docxDe-escalation Case Participant Survey.docxDe-escalation Case Critical Action Checklist.docxDe-escalation Case SP Guide.docx [file mep_2374-8265.11388-s001.zip › E. De-escalation Case SP Guide.docx]

| Appendix E: **De-escalation Standardized Patient Guide** |
| --- |
| This appendix is meant to serve as a guide for the standardized patient (SP). The SP should reference these details in preparation for the case so that this case can be run at its full potential at any institution and with any SP.  We recommend an adolescent-age appearing large male for this case but any gender-identity is adequate if available. Adolescent or young adult SP is ideal to allow credibility for the learners. The SP should be experienced and comfortable with medical simulation cases. Experience with psychiatric emergency scenarios is ideal, though may not be possible.  Please instruct the SP in a shared word or action that they can do to end the sim if they feel the situation is becoming physically or psychological unsafe. |

| **Standardized Patient Script/ Scenario Cues**  Of note, when the learners enter the room, the patient should already be escalated, agitated and/or yelling. | | |
| --- | --- | --- |
| **If/ when….** | **Then….** | **Additional Information** |
| If no one has directly engaged with the patient within 3 minutes… | He will become more disruptive, more agitated and start to yell.  I.e. - “*No one is listening to me, no one cares that I am here. Let me out of here! I just want to go home!”* | If at any point, security is called before the provider tries to verbally de-escalate the patient, the nurse should suggest they try verbal de-escalation first.  Pt is pacing around the room. |
| When the provider makes their first attempt to speak with the patient and asks why they’re upset... | The patient should describe some of the challenges faced by psychiatric patients at that institution.  i.e. - *‘I’ve been in the ER for 20 hours and no one has come to talk to me. I barely got any lunch, the TV was broken, and the kid next to me was screaming for the last hour - wouldn’t YOU be upset? I just want to GO. HOME. Just let me go home!’* |  |
| If the provider is really listening to the patient, and states something that validates them…  *Examples of validating comments:*  *‘Hi, my name is ***. I see that you’re upset. I also see that you are trying to take deep breaths. Thank you for that.’*  *‘I’m sorry that we haven’t had a chance to meet yet. I’m also so sorry that we’re in this situation.’*  *‘I can’t let you go home, but I can bring you a snack or a drink. Have you had anything to eat today? ”* | The patient should reply with something like:  ‘*You’re the first person who has treated me like a human. I just… thank you. But my mind is racing. I’m having a hard time calming down’* | Pacing slows as patient connects with provider.  The provider should attempt to make eye contact with the patient but maintain a safe distance. If the provider gets too close, the patient should become more agitated and start to posture as if they’re going to strike the provider. The patient will calm down as the provider moves away from them and gives them space. |
| If the provider asks the patient what usually helps him calm down… | The patient should state some things that he likes to do, but still remain escalated.  i.e. - ‘*At home, I draw a lot but I doubt I can do that here. I can’t do anything here. This is so STUPID. I want to go home. I can’t even have my own clothes or a pencil, how am I supposed to calm down?’* | If not asked, the parent or provider should offer the information that the patient likes music, drawing, etc.  If the provider says they can provide drawing supplies/ music/ etc. if the patient can demonstrate safe behavior, the patient should agree and start to calm down. |
| If the provider enters the room with multiple people, security guards, etc., or if at any point in the case a crowd of people/ security guards appears… | The patient should become more escalated.  I.e. - ‘*Whoa whoa why are all these people here? You think I’m some kind of animal? Stop surrounding me! Stop looking at me! Get away from me, I’m not even doing anything!’* | Pt yelling, backing away from the guards.  The critical action here is that the provider demonstrates ‘crowd control’ and allows only necessary personnel to be present in order to make it less overwhelming for the patient. |
| If the provider mentions Benadryl to the patient, nurse or their colleague… | The patient should act like they overheard them and get nervous.  I.e. - ‘*No. No Benadryl? Wait, no I think I responded really bad to that last time. Please no. No, not that one’* | Patient visibly anxious, hand wringing, pacing more.  If the patient doesn’t, the nurse or parent should mention that he had a paradoxical reaction to Benadryl last time. |
